# Metachronous splenic metastasis from renal cell carcinoma: a case report

**DOI:** 10.1093/jscr/rjab449

**Published:** 2021-10-22

**Authors:** Rahoui Moez, Boulma Rami, Khouni Hassen

**Affiliations:** Departement of Urology, FSI Hospital, La Marsa, Tunisia; Departement of Urology, FSI Hospital, La Marsa, Tunisia; Departement of Urology, FSI Hospital, La Marsa, Tunisia

**Keywords:** renal cell carcinoma, spleen, metastasis

## Abstract

The causes of isolated solid splenic lesions are wide and varied, and as such can present a diagnostic challenge. Splenic metastases were previously considered exceptionally rare. We report a case of a patient who had isolated splenic metastases with a previous history of left nephrectomy due to a renal cell carcinoma 3 years before. This report represents the first case reported in our country and wants to add to literature one more case of splenic metastasis from renal cell carcinoma.

## INTRODUCTION

Renal cell carcinoma (RCC) accounts for 3% of all cancers [1]. It is the third urologic cancer after prostate and bladder cancers. The preferred sites of metastasis are the lungs, bones, liver and brain [2]; however, splenic metastasis from RCC in extremely rare and up to now only few cases has been described in the available literature. We report the case of a men who presented with a splenic lesion 3 years after radical nephrectomy.

## CASE REPORT

**Figure 1:**
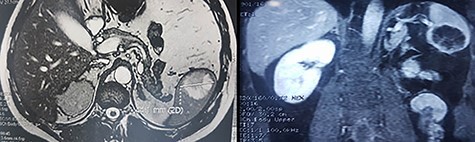
Adominal MRI showing the splenic mass.

**Figure 2:**
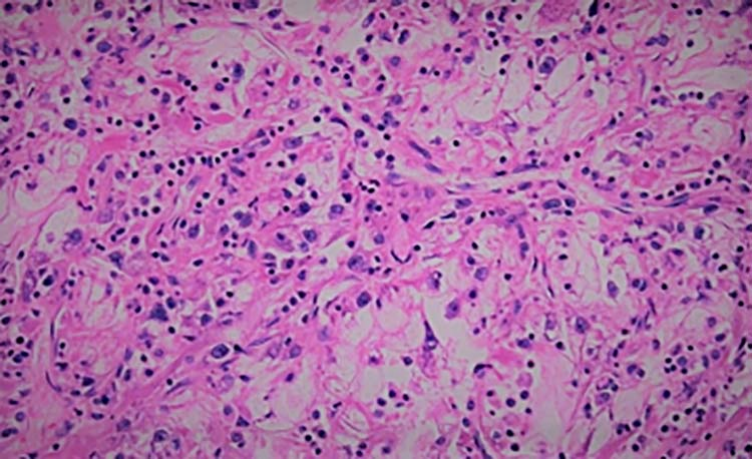
Histology showing clear cell from renal carcinoma.

We report the case of 47-year-old men, with a chronic renal failure, who had been operated on 3 years previously for a total left radical nephrectomy for clear cell renal cancer of stage pT3G1N0M0. During the follow-up of the patient, a routine cancer screening with ultrasound revealed a mass in the spleen of about 4 cm of diameter. Laboratory investigations were normal apart from known renal failure (Table 1). Due to known chronic kidney disease and allergy to Iodinated contrast media, an abdominal magnetic resonance imaging (MRI) confirmed the presence of a splenic lesion (Fig. 1). Based on these radiological findings, the splenic mass was diagnosed as a suspicious metastatic lesion. An open splenectomy was performed. The patient recovered uneventfully and was discharged 5 days after surgery. He received pneumococcal, meningococcal and *Haemophilus influenzae* vaccine. Histologic analysis of the lesion confirmed the presence of clear cell renal cancer metastasis (Fig. 2). The patient was referred to the oncology department for adjuvant treatment with sunitinib. His follow up consisted on abdominal ultrascan (US) every 3 months and MRI at 6 and 12 months from surgery. After 36 months, the patient is doing well with no signs of tumor recurrence.

**Table 1:** Laboratory investigations

Biochemical and hematological parameters	Value
Hemoglobin (g/dl)	12.4
White blood cells	8700
Platelets	278 000
Serum creatinine (umol/L)	175
Calcium (mmol/L)	2.3
Albumin (g/L)	37

## DISCUSSION

Excluding hematological diseases, primary and metastatic tumors to the spleen are uncommon. The reported incidence of metastatic tumors in spleen varies from 0.3% to 7.3%, but it is generally linked to hematological malignancies [2]. Metastasis to the spleen is infrequent. The most common primary cancers are lung cancer, skin malignant melanoma and breast cancer [3]. About 30% of the patients with renal cell carcinoma already have distant metastasis at the time of diagnosis. Splenic metastasis from RCC in extremely rare and up to now only few cases has been described in the available literature [4] (Table 2). The incidence of isolated splenic metastasis from any given primary is also particularly rare. Generally, metastases are asymptomatic and discovered during the follow up with US and computed tomography (CT).

**Table 2:** Summary of cases of isolated splenic metastasis found in the literature

Authors	Age/sex	Metastasing time	Primary	Outcome
Strum	59 y/M	22 y	Right	Dead (5 mo)
Ishida *et al*.	50 y/M	7 y	Left	Alive (6 y)
Nabi *et al*.	50 y/f	(Synchronus)	Left	Alive (6 mo)
Kugel *et al*.	72 y/M	2 y	Left	Dead (1 y)
McGregor *et al*.	65 y/M	(Synchronus)	Left	ND
Shuck-Bello *et al*.	74 y/M	15 y	Right	ND
Ielpo *et al*.	82 y/M	14 y	Left	Alive (1 y 3 mo)
Moir *et al*.	70 y/F	11 mo	Left	Alive (2y)
Nunes *et al*.	60 y/f	5 y	Left	Alive (6 mo)
Hardikar	29 y/M	(Synchronus)	Left	Alive (2 y)
Zhang *et al*.	67 y/M	2 y	Left	Alive (5 mo)
Grewal *et al*.	53 y/m	2 mo	Left	ND
Liu *et al*.	75 y/M	(Synchronus)	Right	Alive (1 y 4 mo)
Ramao *et al*.	48 y/M	11 y	Left	Alive (2 mo)
Rahoui, 2021	47 y/M	3 y	Left	Alive (3 y)

ND: not described.

The definitive diagnosis is based on a histological examination. The cellular diagnosis can be made by using percutaneous biopsy (using US or CT guidance) [5, 6] or endoscopic fine needle aspiration [7]. Surgery for splenic metastasis of kidney cancer is recommended for a palliative purpose and prevention of future complications. In these cases, an adjuvant therapy is recommended but it is effective in only about 10% of patients [8]. The prognosis, if the metastases are multiple is unfavorable, while if it is isolated the surgery, is the best treatment. If a complete resection of the metastasis is achieved the prognosis is favorable [9]. Our patient underwent an open splenectomy without morbidity and was referred to the oncology department for adjuvant treatment with sunitinib. Three years the patient is doing well with no signs of tumor recurrence.

## CONCLUSION

Splenic metastasis from RCC in extremely rare. They are often asymptomatic and generally discovered on surveillance imaging. The optimal treatment is based on splenectomy to avoid any complications. The adjuvant treatment should not be considered a standard of care outside from clinical trials.

## ETHICS APPROVAL AND CONSENT TO PARTICIPATE

The approval of the current study has been granted by the medical committee of research ethics of FSI hospital . Written informed consent was obtained from the patient for publication of this study. A copy of the written consent is available for review by the Editor-in-Chief of this journal on request.

## CONSENT FOR PUBLICATION

Written informed consent was obtained from the patient for publication of this study. A copy of the written consent is available for review by the Editor on request.
